# Monaural auditory spatial abilities in early blind individuals

**DOI:** 10.1177/20416695221149638

**Published:** 2023-02-23

**Authors:** Sara Finocchietti, Davide Esposito, Monica Gori

**Affiliations:** Unit for Visually Impaired People, 121451 Italian Institute of Technology, Genoa, Italy

**Keywords:** audio, space representation, monaural, blindness, localization, bisection

## Abstract

Early blind individuals can localize single sound sources better than sighted participants, even under monaural conditions. Yet, in binaural listening, they struggle with understanding the distances between three different sounds. The latter ability has never been tested under monaural conditions. We investigated the performance of eight early blind and eight blindfolded healthy individuals in monaural and binaural listening during two audio-spatial tasks. In the localization task, a single sound was played in front of participants who needed to localize it properly. In the auditory bisection task, three consecutive sounds were played from different spatial positions, and participants reported which sound the second one was closer to. Only early blind individuals improved their performance in the monaural bisection, while no statistical difference was present for the localization task. We concluded that early blind individuals show superior ability in using spectral cues under monaural conditions.

##  

Nowadays, it is well known that blind people, specifically early blind individuals, namely those blind at birth or that become blind in the first 2 years of age, show a trade-off in auditory localization capabilities. This means that they show superior auditory capabilities in performing some tasks, while they result impaired in others. In particular, they express superior auditory pitch discrimination (Gougoux et al., 2004), map the auditory environment with superior accuracy (Voss et al., 2004), and are more accurate in the monaural localization of eccentric sounds along the horizontal plane (Lessard et al., 1998). On the contrary, the localization of stimuli along the vertical axis (Lewald, 2002; Zwiers et al., 2001), the arm movement reproduction and audio depth discrimination (Cappagli et al., 2017), and the encoding of moving sound sources (Finocchietti et al., 2015; Gori et al., 2017) result impaired. Finally, early blind individuals show a deficit in a specific task that highlights the inexact encoding of Euclidean auditory relationships in this group of individuals: the audio space bisection (Gori et al., 2014), where participants have to estimate the spatial distances among three sounds appearing in sequence, where the first and the third sound are at a fixed position and the second sound appears at any position in between.

To date, it is unclear why early blind people struggle with the audio space bisection task, while in localizing a single sound, they can even outperform the sighted population. Such differences may result from interaction effects among the many acoustic cues available (e.g., binaural cues, monaural spectral cues, dynamic cues, and reverb-related cues (Kolarik et al., 2016; Voss, 2016). However, the comprehension of the interplay among acoustic cues in simple sound localization and space bisection is far from being understood. In this regard, simply attenuating the sounds coming at one ear by means of an ear mold unveiled some interesting effects (Lessard et al., 1998; Van Wanrooij & Van Opstal, 2004; Van Wanrooij & Van Opstal, 2005). For example, one of the pillar studies on auditory localization in visually impaired people showed that some early blind people, under monaural listening, can accurately localize sound sources, regardless of the hemispace in which they are presented, while sighted people cannot (Lessard et al., 1998). A subsequent study compared the neurofunctional activity of early blind and sighted people under monaural listening, identifying a significantly larger activation of the occipital areas in the group of early blind participants whose accuracy was not affected by the ear mold (Gougoux et al., 2005), suggesting that those participants could use monaural spectral cues more proficiently.

Here, we used the same approach, namely performing audio-spatial judgments under binaural versus monaural conditions, to investigate how monaural spectral cues contribute to the final performance in the audio space bisection tasks. First, we tried to replicate the effect found in the seminal work of Lessard et al. (Lessard et al., 1998) by testing sighted and early blind individuals on the single sound localization tasks. We expected a monaural listening-related performance drop in the sighted group but not in the early blind group. Second, we tested the same groups on the audio space bisection task. If monaural spectral cues were involved in the estimation of distances among the bisection's sounds, and early blind individuals were more proficient than sighted in the use of those cues, then the former group's performance drop should be significantly smaller than the latter's under monaural listening, suggesting that they can use spectral cues to infer Euclidean spatial coordinates.

## Methods

### Participants

Eight early blind participants (five females, age range: 26–56, mean age: 37 years old) and eight healthy blindfolded adults (four females, age range: 24–53, mean age: 34 years old) participated in the study.

All the individuals had similar education (at least an Italian high school diploma, indicating 13 years of school). The vision loss of the early blind had different etiology (Table 1). All the individuals had normal hearing (assessed by audiometric test) and no cognitive impairments. The individuals provided written informed consent in accordance with the Declaration of Helsinki. The study was approved by the local ethical committee (ASL 3 Genovese).

**Table 1.** Clinical Details of the Visually Impaired Participants.

S. No.	Gender	Age	Pathology	Residual vision at test
1	M	56	Uveitis	No vision
2	F	32	Retinopathy of prematurity	No vision
3	F	26	Congenital cataract	No vision
4	F	26	Retinopathy of prematurity	No vision
5	M	58	Congenital glaucoma	No vision
6	F	30	Retinitis pigmentosa	No vision
7	F	35	Atrophy of the eyeball	Light and shadows
8	M	33	Leber's amaurosis	No vision

*Note*. The table shows the age at test, the gender, the pathology, and eventual residual vision.

### Procedure and Tasks

**Figure 1.**
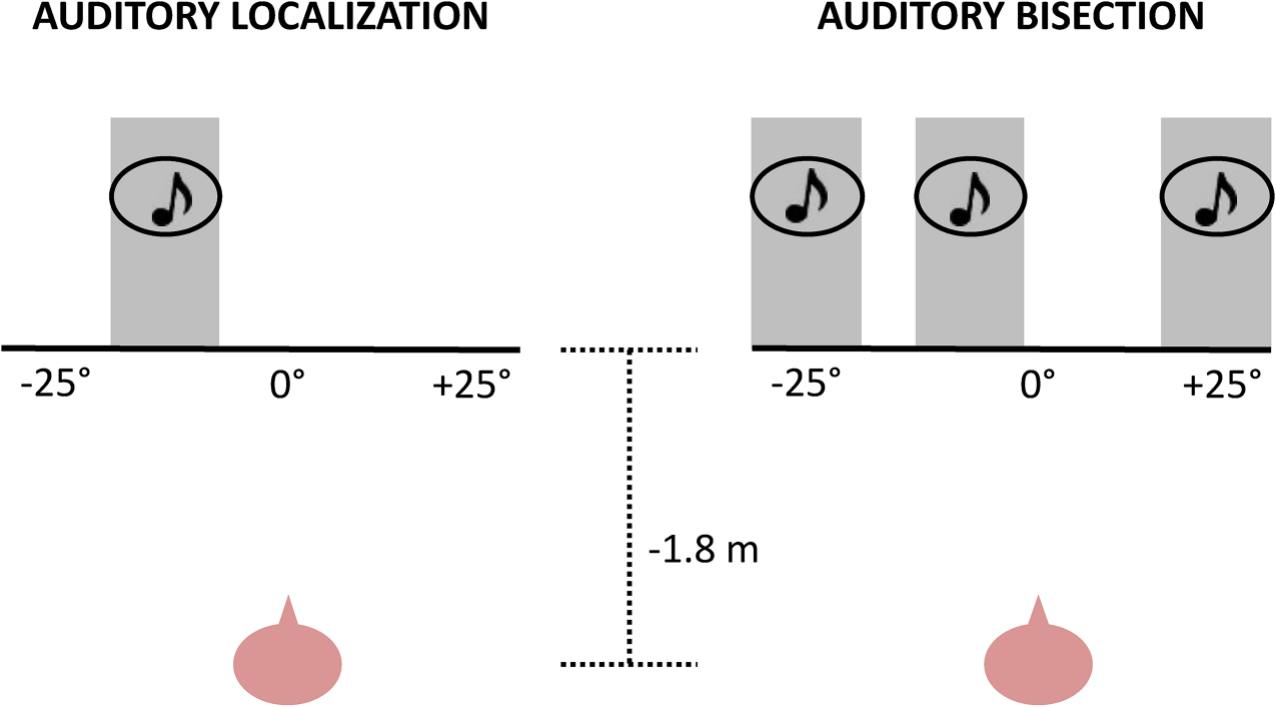
The auditory localization task and auditory bisection task.

The two experimental groups performed two different auditory tasks twice (Figure 1), once monaurally and once binaurally. An array of 23 loudspeakers, arranged in a straight line, positioned between −25° and + 25° (Maxxtro, UK—the speaker array was long 161 cm), delivered auditory stimuli in the form of 500 Hz tones at 70 dB sound pressure level (SPL). In both tasks, the participants were seated in front of the set of loudspeakers, centered in the middle, at a distance of 1.80 m. Their head was free to move, yet they were requested to maintain their head aligned with their sitting position. The tasks were controlled by a custom-designed MATLAB script (MathWorks, USA). Loudspeakers had matching frequency responses, according to the documentation provided by the manufacturer.

#### Auditory Localization Task

For this task, we replicated the procedure as in Cappagli et al. (2017). The participants were holding a cane. A single sound was played from one of the 23 speakers in pseudorandom order. After the audio stimulation, participants pointed to the sound direction with a handheld cane. Pointing positions were then measured by the experimenter and registered.

#### Auditory Bisection Task

For this task, we used the same procedure as indicated by Gori et al. (2014). Three 75 ms stimuli were presented successively at 500 ms intervals, the first at −25°, the third at + 25°, and the second at an intermediate speaker position determined by the QUEST adaptive algorithm (Watson & Pelli, 1983), which estimates the most likely point of subjective equality (PSE), that is, the angle at which the observer's answers are at guess level, after each response, and places the next trial near that estimate. Participants reported verbally whether the second sound was closer to the first or the last sound.

In both tasks, the monaural condition was created as indicated by Lessard et al. (1998): The participant was wearing a soft foam earplug (mean attenuation ¼ 37:5 dB SPL) covered by a hearing protector muff (mean attenuation, 29 dB SPL). The ear cover side was counterbalanced across participants using pseudo randomization. A preliminary test was performed on each participant to ensure that the participant couldn’t perceive sounds on the covered ear: an audio stimulus was presented on the covered side, and the participants were asked if they could hear it.

### Data Analysis and Statistics

For the auditory localization task, localization error was calculated for each individual as their mean absolute error, a common descriptor of error in pointing tasks (Biguer et al., 1984; de Rugy et al., 2000; Schoemaker et al., 2001). In our case, the mean absolute error corresponds to the average of the absolute difference (in centimeters) between the correct position and the position the participant indicated. We considered the correct position as the midpoint of each loudspeaker. The minimum error was 5 cm, which is the distance between the midpoints of two adjacent loudspeakers. The mean absolute error was then converted from centimeters to degrees for each group of individuals. The analyses were then conducted on the mean absolute error in degrees.

**Figure 2.**
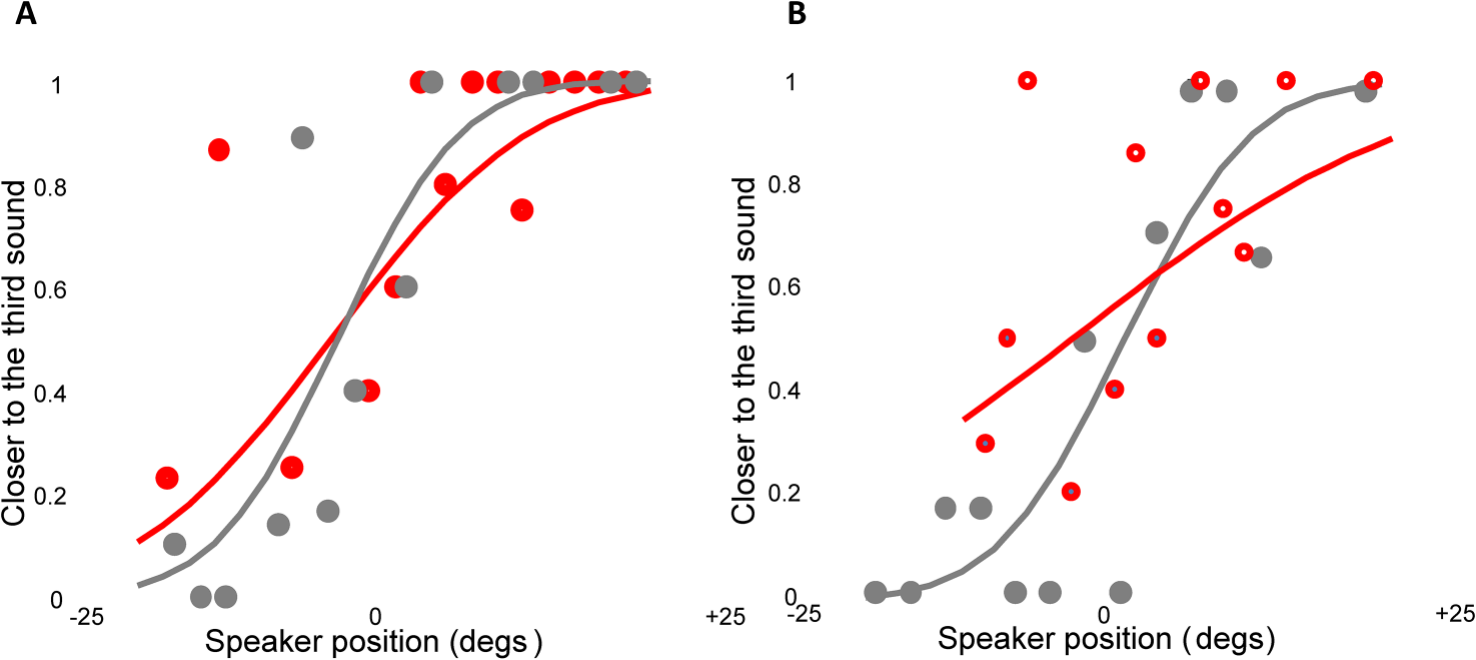
The auditory bisection task, plotting the proportion of “closer to the third sound” as a function of the position of the second sound, averaged separately for the binaural (red symbols) or monaural condition (gray symbols). (A). Sighted blindfolded individuals. (B) Early blind individuals. This figure has been reported for the sake of graphic visualization only. The data analysis has been conducted by fitting the psychometric function to each individual's response set.

Regarding the bisection task, the proportion of trials where the second sound was perceived as closer to the third sound was calculated; then, psychometric curves, in the shape of cumulative Gaussian functions, were fitted on those proportions following a standard psychophysical procedure (Kingdom & Prins, 2010), which consists of fitting the psychometric function to each individual's responses set, extracting individual PSEs and threshold estimates (Figure 2). PSE and threshold estimates were obtained from the mean and *SD* of the fitted psychometric function. Standard errors for the bisection PSE and threshold estimates were calculated by bootstrapping, a technique that takes into account the error associated with each individual threshold as well as the between-subject variance (Efron & Tibshirani, 1994). The obtained PSE and threshold samples were then compared at the group level.

All values are presented as a mean and standard error of the mean (SEM). The Kolmogorov–Smirnoff (KS) test was used to evaluate the normality of the data. Data from each task were then analyzed using a mixed ANOVA between factor *group* (blind, sighted) and within factor *hearing* (mono, binaural). Student *t*-test with Bonferroni corrections were used for post hoc comparisons. The alpha level for effect significance was set to .05.

## Results

All the samples in the dataset resulted normally distributed (KS: *Z* < 0.888; *p* > .200).

**Figure 3.**
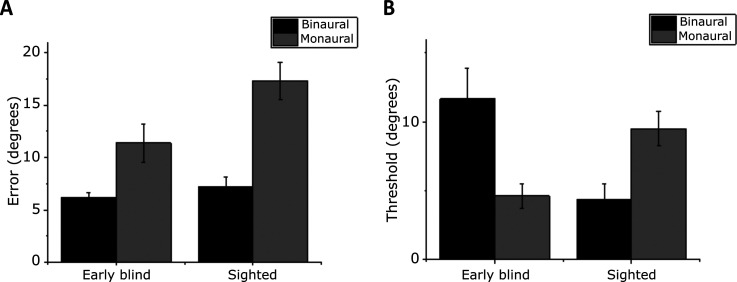
(A) Individual and average localization error relative to the auditory pointing task in early blind and sighted blindfolded individuals, binaurally (black) and monaurally (gray). (B) Average threshold relative to the auditory bisection task in early blind and sighted blindfolded individuals, binaurally (black) and monaurally (gray).

Regarding the ANOVA test for the static localization task, both the main effects were significant (group: *F(1,14)* *=* 7.31, *p* = .017, *η^2^_g_* *=* 0.189; hearing: *F(1,14)* *=* 28.48, *p* < .001, *η^2^_g_* *=* 0.529). The interaction *group* × *hearing* was nonsignificant (*F(1,14)* *=* 2.95, *p* *=* .108, *η^2^_g_* *=* 0.104). As shown in Figure 3(A), the average early blind individuals’ localization error (*M*: 8.80° *95% CI*: [6.68, 10.92]) was smaller (*t(14)* *=* −2.70, *p* *=* .017, *d* *=* −1.35 *95% CI* [−2.43, −0.23]) than that of sighted blindfolded individuals (*M*: 12.27° *95% CI*: [10.10, 14.45]). Moreover, the difference in localization error between binaural and monaural listening regardless of the experimental group (*M*: −7.63 *95% CI* [−10.87, −4.39]) was significantly less than zero ((*t(15)* *=* −5.02, *p* < .001, *d* *=* −1.26 *95% CI* [−1.97, −0.60])), indicating smaller localization error under binaural listening than under monaural listening.

Regarding the ANOVA test for the auditory bisection task, both the main effects were not significant (group: *F(1,14)* *=* 0.69, *p* *=* .422, *η^2^_g_* *=* 0.023; hearing: *F(1,14)* *=* 0.41, *p* *=* .532, *η^2^_g_* *=* 0.015). The interaction *group* × *hearing* was significant (*F*(1,14) = 17.11, *p* = .001, *η^2^_g_* *=* 0.384). As indicated in Figure 3(B), The post hoc between-groups comparisons indicated that in the binaural condition, early blind individuals (*M*: 4.44° *95% CI*: [2.43, 6.44]) performed the task far worse (*t(14)* *=* 2.94, *p* *=* .022, *d* *=* 1.46 *95% CI* [0.33, 2.56]) than sighted blindfolded individuals (*M*: 1.66° *95% CI*: [0.67, 2.66]), while in the monaural condition early blind participants (*M*: 1.76° *95% CI*: [0.0.98, 2.54]) performed much better (*t(14)* *=* −3.20, *p* *=* .013, *d* *=* −1.60 *95% CI* [−2.72, −0.44]) than sighted blindfolded ones (*M*: 3.62° *95% CI*: [2.49, 4.75]). The within-groups comparisons indicated that the early blind individuals’ thresholds were significantly smaller in the monaural condition than in the binaural one (*t(7)* *=* 2.94, *p* *=* .043, *d* *=* 1.04 *95% CI* [0.15, 2.02]), as opposed to sighted individuals (*t(7)* *=* −3.00, *p* *=* .040, *d* *=* −1.06 *95% CI* [−2.05, −0.17]).

## Discussion

This study shows that early blind individuals perform the auditory bisection task better under monaural than binaural listening. In the static localization task, they perform better under binaural than monaural listening. Sighted blindfolded individuals performed both tasks worse under monaural listening.

Concerning the static localization task, the evidence that early blind individuals can reach better performances than sighted individuals is well backed by the literature on the topic (Voss, 2016; Voss et al., 2004; Wan et al., 2010). For example, it is already known that sighted individuals make systematic errors when pointing to sound sources without visual feedback (Vindras et al., 1998), as in our study. The results about the hearing conditions are, instead, more controversial. At first sight, the main effect of *hearing* and the interaction effect not being significant may seem in contrast with the findings Lessard et al. reported (Lessard et al., 1998), namely, that early blind individuals localize sounds under monaural listening better than sighted individuals. In fact, they showed that only some early blind individuals are accurate at localizing sounds under monaural listening as much as under binaural listening, whereas another subgroup of early blind individuals in their sample behaved like the sighted individuals; that is, they exposed an accuracy drop under monaural listening. In our study, the early blind participants were not divided into subgroups according to their performance. In such cases, the group-level performance drop tends toward an intermediate value that depends on the blind group's composition. The ideal experimental design would require assigning early blind individuals to one of the two subgroups beforehand. Unfortunately, the only known predictor for localization task performance is the occipital cortex’s neurofunctional activity during the task itself (Gougoux et al., 2005), which makes the assignment prior to testing currently not possible.

Our results extend the literature about the effect of blindness on audio-spatial abilities by showing that in the audio space bisection task, a specific task that highlights the inexact encoding of Euclidean auditory relationships in this group of individuals (Gori et al., 2014), sighted people showed the expected performance drop under monaural listening, while early blind individuals did not show the drop and even improved their performance. Such a striking effect could be reconducted to the different information content of binaural and monaural cues: binaural cues refer to the discrepancies of inputs between the ears in terms of timing and intensity, whereas monaural cues arise from the spectral filtering of sounds spontaneously occurring when the sound waves interact with the human (upper) body (Van Wanrooij & Van Opstal, 2004). One of the reasons why the early blind participants showed supra-normal performance might be that they utilize auditory spectral cues more effectively, as suggested by Doucet (Doucet et al., 2005). In his previous study, he manipulated the ability to use spectral cues in early blind individuals and found a significant increase in localization errors when their ability to use spectral cues was altered. The hypothesized group-related difference in processing spectral information is coherent with our pattern of results showing the performance improvement under monaural listening in the spatial bisection but not in the static localization. Indeed, spectral cues are more helpful for discriminating peripheral sources (Voss et al., 2011), meaning that the spectral cue captured from a single ear could be more informative for properly locating the first and the third sound and consequently helping in defining the Euclidean distances between the three sounds proposed. Likewise, integrating the spectral information from both ears might require some calibration to be properly processed. Such calibration may arise from visual experience (Gori et al., 2014) or, when vision is absent, other compensatory channels, for example, audio-motor associations (Esposito et al., 2021). An additional factor supporting this theory is that blind echolocators, namely people trained to detect objects in their environment by sensing echoes from those objects, can be as good as sighted blindfolded controls in the auditory bisection task (Vercillo et al., 2015).

One important matter of discussion concerns the difference in effect size and direction within the early blind group in the two tasks. One may argue that such difference reflects a different contribution of monaural and binaural acoustic cues to the spatial reasoning strategies the brain uses to encode audio-spatial information (Gori et al., 2014; Rabini et al., 2019; Voss, 2016). Indeed, it has been shown that the brain encodes spatial information following mainly two strategies: the egocentric strategy, where spatial information is observer-dependent, and the allocentric strategy, where spatial information is observer-independent (Klatzky, 1998). However, whereas the sound localization task requires the use of an egocentric strategy by design, the audio space bisection used here can be performed using an egocentric strategy, such as comparing each of the three sound positions with the prior knowledge of the straight-ahead direction (Odegaard et al., 2015), as well as using an allocentric strategy, such as directly mapping the distances among sounds without relying on egocentric references. Simple tricks may be used to resolve the task's ambiguity, such as introducing a random offset shared among the three sounds to prevent the use of egocentric strategies (Rabini et al., 2019). However, the setup employed here does not allow for such an experimental manipulation, for the first and the third sounds have already been placed at the speaker array's ends.

The findings reported in the present experiment are limited to the azimuth: elevation and depth have not been explored. However, contrarily to azimuthal localization, binaural cues play a secondary role in elevation and depth estimation; therefore, monaural and binaural spectral cues may work differently in different dimensions, as well as in different populations. As a matter of fact, the use of a straight speaker array instead of a circular one may have introduced a confounding interaction effect between spectral cues for azimuth and depth estimation, as the central speakers and the peripheral speakers have different distances from the observer. Indeed, it has been shown that early blind and sighted individuals have different audio distance estimation skills (Kolarik et al., 2016; Voss, 2016). We acknowledge such confounding factor; however, we deem it negligible since the difference in distance from the listener between external and central speakers (10 cm) is 5.5% the distance under judgment (180 cm), a value well below the intra-individual variability range for audio distance estimation, which can be as large as 20% to 60% the distance under judgment (Anderson & Zahorik, 2014; Kolarik et al., 2016). This means that, in all probability, the difference in distance was unperceivable.

In conclusion, early blind individuals perform complex audio-spatial tasks requiring a metric representation of space in the horizontal plane better with monaural cues than with monaural and binaural cues. This could be due to the superior ability to use spectral cues in monaural conditions. This result provides important information for developing tailored rehabilitation programs for visually impaired people. For example, it suggests that spectral cues can be used to train the brain to properly integrate the spectral information received by the two ears and consequently improve the binaural performance of spatial tasks that require a Euclidean representation of space.
